# Supplementary material for: Unconditionally teleported quantum gates between remote solid-state qubit registers
Source: Nat Commun. 2026 May 26;17:4694. doi: 10.1038/s41467-026-72818-6 (PMC13212882; doi:10.1038/s41467-026-72818-6)
Supplement: Supplementary file 1 — Supplementary Information [file 41467_2026_72818_MOESM1_ESM.pdf]

# Supplementary Information for "Unconditionally teleported quantum gates between remote solid-state qubit registers"

Mariagrazia Iuliano[1], Nicolas Demetriou[1], H. Benjamin van Ommen[1], Constantijn Karels[1], Tim H. Taminiau[1], Ronald Hanson[1*]

[1]QuTech & Kavli Institute of Nanoscience, Delft University of Technology, Delft, 2628 CJ, the Netherlands.

*Corresponding author(s). E-mail(s): r.hanson@tudelft.nl;

## Supplementary Note 1 Experimental setup & Operations

The experimental setup for Alice and Bob is similar; hence a single common description is provided here. The NV center platform is composed of a type IIa chemical vapor deposition diamond, cut along the ⟨111⟩ crystal orientation (Element Six), where Solid Immersion Lenses are fabricated around single defects to improve the collection efficiency together with anti-reflection coating. Lithographically deposited gold on the diamond surface acts as a stripline for microwave, DC voltage and radio-frequency delivery. The sample is mounted on a PCB and placed on a sample holder in a closed-cycle cryostat (Montana Cryostation). In the back of the sample holder, a static neodymium magnet is inserted. Additional magnets for magnetic field alignment purposes are placed outside the sample chamber at room temperature. Optical access to the diamond sample is obtained with a room temperature confocal microscope objective that is mounted on a three-axis piezo stage. A detailed schematic of the optics used for excitation and collection can be found in Ref. [1].

The negatively-charged state of the NV-center is a spin-1 system, whose ground state is fully non-degenerate in the presence of an external magnetic field [2]. In Supplementary Figure 1, we include a schematic of the optical and microwave transitions that are relevant for this work. To achieve photon indistinguishability, a DC voltage

(range ±15 V) is applied to exploit the DC Stark effect that effectively tunes the optical transitions and brings the transition m$_s$=0→ $E_{x/y}$ of the two nodes in resonance with each other at 470.4550 THz. Spectral wandering over time is compensated by a Proportional-Integral-Derivative control loop on the applied DC voltage, whose error signal is computed on the average photon counts during the Charge-Resonance check.

The excitation of the optical transitions stimulates the NV to emit single photons (Zero-Phonon Line) or photons+phonons (Phonon-Side Band) according to the Debye-Waller factor. The ZPL photons are used to generate remote entanglement. Using narrow-band filters and cross-polarization techniques, the ZPL photons are separated from the PSB and the excitation light and directed towards the midpoint. Photon detection is achieved via Superconducting Nanowire Single Photon Detectors (PhotonSpot), connected to the output ports of a 50:50 (effectively measured 45:55) in-fiber beam splitter, and show a dark count rate ≤1 Hz each. The PSB is used to read out the qubit state in single-shot mode by state-dependent excitation and discriminating on whether zero or non-zero PSB photons were detected. The detection is achieved using an Avalanche PhotoDiode at each node (Laser Components, Count FC 10C/20C) that shows a dark count rate of 15 Hz for Alice and 6 Hz for Bob.

The transitions denoted with "Reset" are used to initialize the qubit state in $|0\rangle$. In Alice, these two transitions are separated by 480 MHz, hence we use two separate red lasers (Toptica TA-SHG and DL Pro) to address each one of them and achieve an efficient reset process. For Bob, the separation is efficiently covered by the power broadening of a single laser pulse parked in the middle of the two transitions.

To keep the NV in the desired charge state ($NV^-$), a recharging mechanism is needed. In this case, we exploit a two-photon process when addressing the ZPL transition of the neutral charge state ($NV^0$) [3], that deterministically ionizes to $NV^-$. For this we use a single laser per node around 575nm (Toptica DL-SHG pro). For the high-magnetic field setup, Alice, the frequency splitting between the relevant $NV^0$ transitions is ∼200MHz [4], and also in this case we exploit the power broadening of the pulse to effectively address both transitions simultaneously. Typical power values utilized for effective recharging are 400 nW (30 nW) for Alice (Bob) for hundreds of $\mu$s. Alternatively, two laser pulses, each one on resonance with one spin-selective transition, is a valid method for efficient recharging. However, it comes at the cost of a more complex setup, requiring frequency locking and frequency modulation methods of two laser pulses.

The single-qubit gate on the electron spin state is performed by applying microwave pulses to the spin transitions denoted with the purple cycle in Supplementary Figure 1. The microwave signals' source is provided by the R&S SGS100A and is IQ-modulated via the Zurich Instruments HDAWG. The signal is amplified up to 42 W (20 W) (AR 40S1G4) before reaching the sample for Alice (Bob).

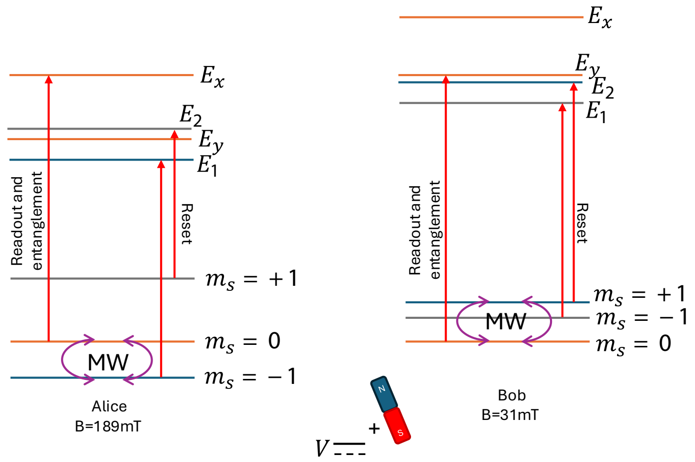


**Fig. 1** Energy level diagram (not in scale). Alice and Bob are biased at different magnetic fields resulting in different energy splittings in the electronic ground state. In Alice's case, the $m_s$=-1 state crossed the $m_s$=0 state and becomes the lowest energy state. The crossing happens at 100mT. The excited states are tuned via an external DC field, ensuring that the $m_s$=0→E$_x$ transition of Alice has the same frequency as $m_s$=0→E$_y$ of Bob.

The HDAWG is used for nanosecond-precision signals and part of the experimental logic. On a higher level, the experimental sequences and logic are orchestrated by a multi-module microcontroller unit (Jäger ADwin-Pro II T12), including the classical information exchange between the nodes via the TiCo module.

For the DDRF method, the RF signal is generated by the HDAWG and amplified before being mixed with the microwave signal and delivered to the chip. The RF signal is a square pulse, whose rise and fall transitions incorporate a $\sin^2(t)$ signal to reduce transient oscillations. Experimental details and theoretical considerations on the DDRF method can be found in Ref. [5].

# Supplementary Note 2 Calibration routine

Before being able to run the experiments described in the main text, it is necessary to prepare the setup and calibrate the relevant parameters for each qubit. In this section, we describe the general calibration routine, differentiating whether the calibration is targeted at the physical setup, at the electron spin qubit or the nuclear spin qubit. The calibration routine and experimental sequences are backed up by the QMI software package [6].

## Supplementary Note 2.1 Setup calibration

The setup calibration starts with the calibration of the laser power levels, by sweeping the amplitude of the RF tone that drives the acousto-optical modulators for each laser and detecting with a power meter the corresponding laser power at the setup.

Subsequently, we calibrate the position of the microscope objective with respect to the emitter. To do so, we use green laser photoluminescence, collecting the emitted PSB photons when scanning the objective position along the three axes.

Other relevant setup calibrations regard the cross-polarization alignment to ensure that the laser photons are properly rejected in the ZPL collection path. For this, a set of automatized half and quarter waveplates placed in the ZPL path is scanned. The optimal position corresponds to the minimum amount of photon count rate in the SNSPDs when the resonant red laser is on. A similar procedure is followed to enable homodyne interference between the two setups at the mid-point in the global phase stabilization scheme. Namely, a motorized half-waveplate at each setup ensures that the same amount of coherent light is sent from each node to the midpoint. Details on the phase stabilization setup and procedures are explained in Ref. [1].

## Supplementary Note 2.2 Electron spin calibration

Provided that the NV is in the right charge state and the laser frequencies are on resonance with the relevant transitions (validated via the Charge-Resonance check), the calibration starts with the microwave pulses for the single-qubit gates on the communication qubit. The summary of the calibrated parameters with their typical values is inserted in Table 1. To obtain an arbitrary rotation along a specific axis ($\alpha$-pulse), apart from the $\pi/2$ rotation, we use the same duration of the $\pi$ pulse and reduce the amplitude accordingly to the desired angle of rotation.

Next, the routine focuses on the calibration of the remote entanglement generation

| **Parameter** | **Alice** | **Bob** |
|---|---|---|
| Frequency | 2.414GHz | 3.733GHz |
| Power | 42W | 20W |
| $\pi$ Duration | 215ns | 205ns |
| $\pi$ Amplitude (fraction) | 0.88 | 0.94 |
| $\pi$ Skewness | $9.85{\cdot}10^{-9}$ | $-3.34{\cdot}10^{-9}$ |
| $\pi$: $P(\|0\rangle)$ after 7 pulses | 4% | 0.5% |
| $\pi/2$ Duration | 150ns | 135ns |
| $\pi/2$ Amplitude (fraction) | 0.40 | 0.52 |
| $\pi/2$ Skewness | $1.28{\cdot}10^{-8}$ | $-5.24{\cdot}10^{-9}$ |
| $\pi/2$: $P(\|0\rangle)$ after 6 pulses | 2% | 0.5% |

**Table 1** Relevant parameters for the calibration of the microwave pulses. The microwave pulse shape is a skewed Hermite pulse. The amplitude is reported as a fraction of the maximum output voltage of the IQ modulation channels.

parameters, which are summarized in Table 2, additionally including the optical phase stabilization and the entangled state phase measurement, whose values change over time due to setup alignment and ambient conditions.

| Parameter | Alice | Bob |
|---|---|---|
| Counts per shot $p$ | $0.9{\cdot}10^{-4}$ | $1.8{\cdot}10^{-4}$ |
| Bright state population $\alpha$ | 0.06 | 0.03 |

| | |
|---|---|
| Entanglement attempt duration | 8.392$\mu$s |
| Detection window | 7ns |

**Table 2** Remote entanglement relevant parameters. The $\alpha_B$ and $p$ parameters reported are typical values, as they can fluctuate based on external conditions. Particularly, $\alpha_B$ is set to fulfill the expression $p_A\alpha_A = p_B\alpha_B$. The ratio $\alpha_B/\alpha_A$ is in the range 0.5$\pm$0.1.

## Supplementary Note 2.3 Nuclear spin calibration

The third part of the calibration focuses on the data qubit, namely the control of single nuclear spins. Despite the use of two different methods for the control, the routine is very similar. The preliminary step is to identify a well-isolated $^{13}$C. In the case of DD method, this is obtained by sweeping the interpulse delay in a repeated XY8 sequence, when the electron spin is initialized in a superposition state. Interpulse delays $\tau$ that are on resonance with a single nuclear spin result in a coherent inversion of the electron spin state [7]. For the selected nuclear spin, we obtain $\tau$=12.452$\mu$s. For the DDRF method, we sweep the frequency $\omega_{RF}$ of the RF field, while the repeated XY8 sequence has a fixed interpulse delay of $\tau$=21.8$\mu$s.

Once the target nuclear spin is selected, we can calibrate the conditional and unconditional gates. This is achieved by tuning the amount of XY8 pulses. For the DD case, we obtain $N^{DD}_{cond}$=48, while for the DDRF the number of pulses can be tuned for time and synchronization reasons by changing the amplitude of the driving RF field, with an upper limit set by heating.

As the electron spin dynamics affect the precession of the nuclear spin due to the hyperfine interaction, it is necessary for effective control to characterize these precession frequencies. For the DD method (Bob), these frequencies can be extrapolated from a detuned Ramsey-type experiment using the nuclear spin initialized in a superposition state and the electron spin in an eigenstate. From fitting the data, we can extrapolate the frequencies and the $T_2^*$ value. The values of the precession frequencies for the two possible electron spin eigenstates are then used to calculate the phase that the nuclear spin state picks up under the electron spin dynamics, provided that it is known how much time the electron spin spends in such states.

In the case of DDRF (Alice), we use a Ramsey-type experiment with electron spin in $|0\rangle$ to determine the precession frequency $\omega_0$ of the nuclear spin around the $\hat{z}$ axis. When the electron spin is in $|1\rangle$, the nuclear spin is driven by the RF field in the $\hat{x}$-$\hat{y}$ plane. The Ramsey experiments are shown in Supplementary Figure 2, while the results are summarized in Table 3.

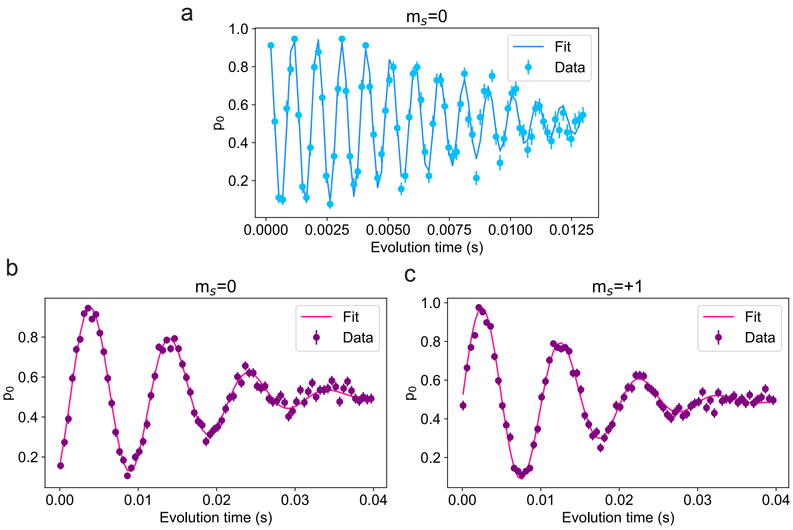


**Fig. 2** Ramsey measurement. a) Experiment for Alice nuclear spin when the electron spin is initialized in $|0\rangle$. From the fit, we obtain $\omega_0$=2.021 MHz. b) Experiment for Bob nuclear spin with the electron spin in $|0\rangle$. The resulting frequency is $\omega_0$=327.1 kHz. In c) we report the experiment when the electron spin is in $|1\rangle$, resulting in $\omega_1$=355.6 kHz. Error bars represent one standard deviation.

| Node | $A_{\parallel}$ ($\times 2\pi$) | $A_{\perp}$ ($\times 2\pi$) | $T_2^*$ (avg) | $\omega_0$ | $\omega_1$ |
|---|---|---|---|---|---|
| Alice | -30.0 kHz | $\sim 0$ | 9.4(4)m s | 2.021 MHz | $\omega_{RF}$=2.051 MHz |
| Bob | 28.2 kHz | 11.9 kHz | 19.6(5) ms | 327.1 kHz | 355.6 kHz |

**Table 3** Nuclear spin characteristic parameters. For DDRF, $A_{\perp} \ll \omega_{RF}$, hence we neglect this term. In the reference frame of the RF field, $\omega_0 = A_{\parallel} = \omega_L - \omega_{RF}$ and $\omega_1 = 0$. The relative error on the obtained frequencies is 0.5% for Alice and 0.2% for Bob [8].

# Supplementary Note 3 Nuclear spin phase evolution during entanglement attempts

A separate discussion is needed on the evolution of the nuclear spin during network activity. During an entanglement attempt, and specifically during the reset pulse, the electron spin state undergoes a stochastic process, given that the exact moment when it flips back to $|0\rangle$ is probabilistic. From the nuclear spin perspective, this results in a dephasing mechanism. As reported in the main text, the phase that the nuclear spin acquires during the entanglement attempts needs a proper separate calibration. The resulting phase per entanglement attempt can be seen as an average phase due to the stochasticity of the process. To calibrate such a phase, we follow two different protocols due to the use of two different control techniques.

For the DDRF setup (Alice), the calibration process is faster, since the phase acquired during the entanglement attempt is fed to the local oscillator of the RF

field and used to update the phase of the next RF pulse. We first characterize a pre-entanglement global phase that ensures that without any entanglement attempts, the nuclear spin is correctly rephased for the readout measurement. This phase is independent of the initial state of the nuclear spin, so for consistency, we initialize it in the $|X\rangle$ state. Subsequently, we can characterize the single entanglement attempt phase. To do so, we initialize the nuclear spin state in $|X\rangle$, sweep the number of entanglement attempts (e.g. from 1 to 25), and then measure in the X basis. Given that the local RF oscillator was not updated, we obtain a sine-type signal, from which we can extract the average phase for a single entanglement attempt. A typical value for the phase of a single attempt is $54^\circ$.

For the DD setup (Bob), the rephasing is executed via a tailored XY8 sequence. The calibration of such sequences comprises several steps. First of all, we compile a table of interpulse delays for the XY8 sequence where we ensure that no coupling to surrounding nuclear spins is involved (1% tolerance on the electron spin coherence loss). A typical range for the interpulse delay is [$2.8\mu$s-$3.2\mu$s]. The next step includes finding the optimal rephasing interpulse delay for a certain number of entanglement attempts. For this, we first initialize the nuclear spin in $|X\rangle$, we sweep the number of entanglement attempts (e.g. from 1 to 10) and for each number of entanglement attempts we sweep the total duration of the rephasing XY8 sequence by using the precompiled table of optimal interpulse delays, and finally we measure the nuclear spin in the X basis. For each number of entanglement attempts, we obtain a sine-like signal over the interpulse delays. We jointly fit these curves by imposing the same frequency as a fit parameter, and from that we extract the phase acquired for each number of entanglement attempts. In the next step, we fit the obtained phases with a linear function to extrapolate the general phase rule for $N$ number of entanglement attempts, bounded between 0 and $2\pi$. We then convert the phase into an XY8 duration knowing the evolution frequency, and we compile the corresponding interpulse delays, chosen among those that pass the non-coupling check, into a look-up table in the HDAWG that can be used in real-time during the experiment. In this method, the main source of errors comes from the fit error and from the necessity of using a discrete set of XY8 durations, while for the DDRF method the only source of error is the curve fit.
These methods can be extended to a larger number of entanglement attempts by repeating the procedure for a batch of higher number of entanglement attempts (e.g. from 51 to 75 to infer the phase up to 100 entanglement attempts). In this way, we can contain the cumulative error on the phase caused by the probabilistic contribution to the phase and by the curve fitting error. In case the single entanglement attempt duration is longer, for example if we want to extend the demonstration to a long distance scenario, the cumulative phase error is the same, as with longer entanglement attempts we only change the deterministic contribution, provided that the optical reset pulse has the same duration and amplitude.

# Supplementary Note 4 Readout correction on nuclear spin state mapping

As illustrated in the main text, the readout of the nuclear spin state is assisted by mapping such a state into the electron spin state. Hence, when reading out, infidelity is caused by the known tomography errors during the single-shot readout of the electron spin and the errors that occur during the mapping of the nuclear spin state into the electron spin state. To estimate and correct for the latter, we adopt a combination of the strategies reported in Refs. [9, 10]. During the mapping, the electron spin is subjected, among other sources of errors, to dephasing that is faster than the optimal read-out time, measured in number of microwave pulses $N_{RO}$ necessary to complete the mapping. To characterize this dephasing, we perform the experiment displayed in Supplementary Figure 3a, during which the target nuclear spin is left uninitialized, but the interaction with the electron spin is activated via the repeated XY8 sequence similar to that used for the electron-nuclear conditional gate. Hence, in the case of Alice, this is also interleaved with RF pulses. The result is a damped oscillation in the number of XY8 repetitions due to repeated entangling and disentangling of the electron with the nuclear spin, displayed in Supplementary Figures 3a-b. Doing the calibration this way we avoid the introduction of additional errors due to the initialization process of the nuclear spin state, which is also assisted by the electron spin, separating the readout sequence from it. An imperfect initialization process can, in principle, lead to correct readout results, as the mapping process is not symmetric and, therefore, the readout might compensate for incorrect initialization and, at the same time, generate a correlated error on the electron spin, obscuring the dephasing only given by the readout. We fit this curve to the function:

$$\langle\sigma_y^e\rangle(x, \delta, d, N_0, \beta) = \delta \exp[-(x/N_0)^d] \cos(\beta x) \tag{1}$$

in which $\delta$ represents the maximum contrast achieved by the signal, $N_0$ and $n$ characterize the exponential decay due to the dephasing of the electron spin; the cosine function represents the oscillating behaviour that the signal should have under perfect conditions. The parameter $\beta$ refers to the electron-nuclear coupling. From this, it is possible to extract the correction $C_{en}$ defined as:

$$C_{en} = \delta \exp[-(N_{RO}/N_0)^d] \sin(\beta N_{RO}) \tag{2}$$

that we use to rescale the single-shot readout corrected expectation values obtained from the electron-assisted nuclear spin tomography as $1/C_{en}$. We obtain correction factors of $1/C_{en}^{Alice}$=1.08(2) and $1/C_{en}^{Bob}$=1.05(3).

# Supplementary Note 5 Experiment simulations

The simulated outcomes of the two experiments can be found at [8]. For the simulation of the remote entangled state, the simulation is adapted from [11].
The GHZ experiment simulation includes errors from the dephasing on the data qubits

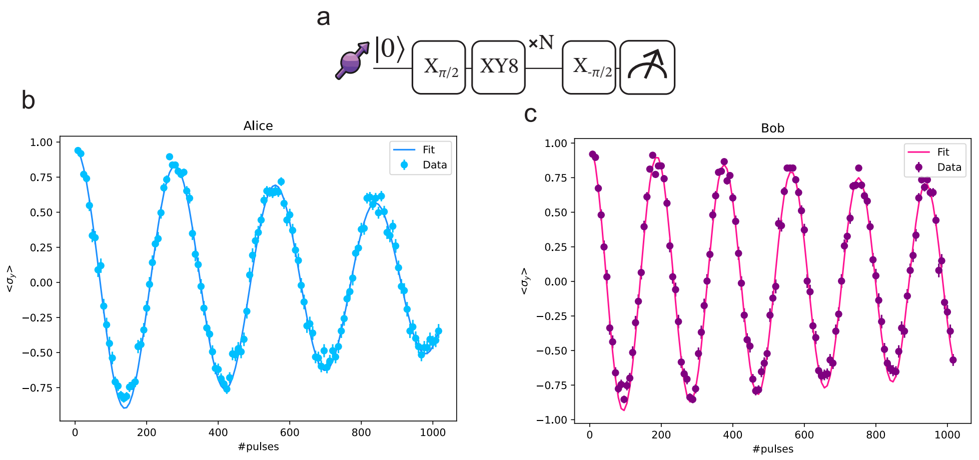


**Fig. 3** Nuclear spin readout error characterization. a) Experimental sequence executed on the electron spin to isolate the assisted-readout errors from any nuclear spin initialization imperfections. For Alice, the inter-pulse delay is filled with RF pulses on resonance with the target qubit. b) and c) display the recorded signal and the corresponding fit to extract the relevant parameters for the computation of the correcting factors. In b), we obtain the following parameters from the curve fitting: $\delta_{Alice}$=0.95(1), $N_0^{Alice}$=1442(97), $d_{Alice}$=1.2(1), $\beta_{Alice}$=0.0224(3). For c) we obtain: $\delta_{Bob}$=0.98(2), $N_0^{Bob}$=3495(954), $d_{Bob}$=0.9(2), $\beta_{Bob}$=0.0334(2). Error bars represent one standard deviation.

from the generation of the remote entangled state; the depolarization of the communication qubits after entangling with their local data qubit; the dephasing on the data qubit caused by wrong readout assignment of the communication qubit.
The simulation for the non-local C-NOT gate includes the same errors of the GHZ case, with the exception of the last dephasing error, which is substituted with the incorrect feedback operation on the data qubit corresponding to the incorrect readout assignment probabilities.

# Supplementary Note 6 Data acquisition

The setup can be fully operated remotely. For the two network experiments, data are acquired in batches of 1 hour, interleaved with partial calibration of the setup. The partial calibration is focused on the entanglement generation parameters, particularly the measurement of the phase of the entangled state and the optimal cross-polarization point. These parameters are affected by small drifts in the optical setup that are mainly due to the degradation of the vacuum of the sample chamber (leading to the formation of layers of ice), as well as due to vibrations, temperature and humidity fluctuations of the laboratory.

The average experimental rate is in the range of (23-42)mHz, with a total number of data points of: 360 for the GHZ experiment, 400 for the classical truth table of the C-NOT gate and 234 points for the creation of the remote entangled state via the non-local C-NOT gate. The variation in the experimental rate is due to the

daily fluctuations in counts per shot of the two NVs, which directly affect the rate of entanglement generation, and to the charge fluctuations due to the DC Stark tuning, which affect the number of CR checks required to bring both nodes on resonance with each other, increasing experiment overhead time. Besides the rate, such fluctuations directly affect the maximum achievable fidelity. During entanglement generation, for all experiments, we keep the bright state population parameter $\alpha_A$ of Alice fixed at 0.06, while $\alpha_B$ of Bob is adapted to fulfill the equality $p_A\alpha_A = p_B\alpha_B$. This leads to an entanglement generation probability of $1.1 \cdot 10^{-5}$. However, during the experiment, such conditions might not be fulfilled at all times. The simulations do not take this variation into account. On the other hand, variations in the DC field necessary to keep both nodes at the same resonance frequency during the entanglement attempts affect the overall indistinguishability of the single photons, and therefore the fidelity.

# References


[1] Pompili, M. *et al.* Realization of a multinode quantum network of remote solid-state qubits. *Science* **372**, 259–264 (2021). URL https://www.science.org/doi/10.1126/science.abg1919. Publisher: American Association for the Advancement of Science.

[2] Doherty, M. W., Manson, N. B., Delaney, P. & Hollenberg, L. C. L. The negatively charged nitrogen-vacancy centre in diamond: the electronic solution. *New Journal of Physics* **13**, 025019 (2011). URL https://iopscience.iop.org/article/10.1088/1367-2630/13/2/025019.

[3] Siyushev, P. *et al.* Optically Controlled Switching of the Charge State of a Single Nitrogen-Vacancy Center in Diamond at Cryogenic Temperatures. *Physical Review Letters* **110**, 167402 (2013). URL https://link.aps.org/doi/10.1103/PhysRevLett.110.167402.

[4] Baier, S. *et al.* Orbital and Spin Dynamics of Single Neutrally-Charged Nitrogen-Vacancy Centers in Diamond. *Physical Review Letters* **125**, 193601 (2020). URL https://link.aps.org/doi/10.1103/PhysRevLett.125.193601.

[5] van Ommen, H. *et al.* Improved Electron-Nuclear Quantum Gates for Spin Sensing and Control. *PRX Quantum* **6**, 020309 (2025). URL https://link.aps.org/doi/10.1103/PRXQuantum.6.020309. Publisher: American Physical Society.

[6] Raa, I. T. *et al.* QMI - Quantum Measurement Infrastructure, a Python 3 framework for controlling laboratory equipment (2023). URL http://doi.org/10.4121/6D39C6DB-2F50-4A49-AD60-5BB08F40CB52.

[7] Taminiau, T. H. *et al.* Detection and Control of Individual Nuclear Spins Using a Weakly Coupled Electron Spin. *Physical Review Letters* **109**, 137602 (2012). URL https://link.aps.org/doi/10.1103/PhysRevLett.109.137602.

[8] Iuliano, M., Demetriou, N., Van Ommen, H. B., Taminiau, T. H. & Hanson, R. Data underlying the publication "Unconditionally teleported quantum gates between remote solid-state qubit registers" (2025). URL https://doi.org/10.4121/a33310e8-a19b-4aac-8057-37ab1363e42e.v1.

[9] Cramer, J. *et al.* Repeated quantum error correction on a continuously encoded qubit by real-time feedback. *Nature Communications* **7**, 11526 (2016). URL

https://www.nature.com/articles/ncomms11526.

[10] Randall, J. *et al.* Many-body–localized discrete time crystal with a programmable spin-based quantum simulator. *Science* **374**, 1474–1478 (2021). URL https://www.science.org/doi/10.1126/science.abk0603.

[11] Hermans, S. L. N. *et al.* Qubit teleportation between non-neighbouring nodes in a quantum network. *Nature* **605**, 663–668 (2022). URL https://www.nature.com/articles/s41586-022-04697-y.
